# Photosensitizers in prostate cancer therapy

**DOI:** 10.18632/oncotarget.15496

**Published:** 2017-02-18

**Authors:** Taher Gheewala, Troy Skwor, Gnanasekar Munirathinam

**Affiliations:** ^1^ Department of Biomedical Sciences, University of Illinois, College of Medicine, Rockford, IL, USA; ^2^ Department of Chemical and Biological Sciences, Rockford University, Rockford, IL, USA

**Keywords:** prostate cancer, photodynamic therapy, photosensitizers

## Abstract

The search for new therapeutics for the treatment of prostate cancer is ongoing with a focus on the balance between the harms and benefits of treatment. New therapies are being constantly developed to offer treatments similar to radical therapies, with limited side effects. Photodynamic therapy (PDT) is a promising strategy in delivering focal treatment in primary as well as post radiotherapy prostate cancer. PDT involves activation of a photosensitizer (PS) by appropriate wavelength of light, generating transient levels of reactive oxygen species (ROS). Several photosensitizers have been developed with a focus on treating prostate cancer like mTHPC, motexafin lutetium, padoporfin and so on. This article will review newly developed photosensitizers under clinical trials for the treatment of prostate cancer, along with the potential advantages and disadvantages in delivering focal therapy.

## INTRODUCTION

Prostate cancer (PCa) is the second most common cancer in men. In 2016, the American Cancer Society has estimated 180,890 new cases of PCa in the United States alone resulting in 26,120 deaths [1]. Current treatment options, although effective, pose severe side effects that impact the quality of life of patients. As screening procedures have become more aggressive and accessible, it has aided in screening and diagnosis of early stage PCa [2]. Radical therapies available for organ confined cancer involve physical or chemical castration. Active surveillance, or delayed selective intervention for men with organ confined low risk PCa, has given rise to an interest in focal therapy, which can be organ-sparing. Focal therapy is aimed at treating clinically relevant volumes of cancer within the gland while leaving other areas untreated. A number of modalities have been identified to deliver such treatment like high-intensity focused ultrasound [3], cryotherapy [4], and photodynamic therapy (PDT). The first use of PDT in a clinical setting for PCa used two tissue based photosensitizers (PS) in two patients, where one received a hematoporphyrin derivative and the other received Photofrin. The 3 month biopsy of these two patients showed neither had residual disease [5]. Another study assessed the uptake of ALA in PCa in six patients. Fluorescence microscopy showed ALA had been taken up in the cancer cells sparing the surrounding tissue [6]. However, prolonged skin photosensitivity and less tissue penetration of short wavelength light are common problems associated with first generation PS. The formation of aggregates of the PS inside of cells, leads to increased susceptibility to prolonged phototoxicity. This has led to the development of second and third generation PS, which are eliminated from the body within hours of administration. This review is aimed at discussing such photosensitizers that are in clinical trials for the treatment of PCa.

## PHOTODYNAMIC THERAPY

**Figure 1:**
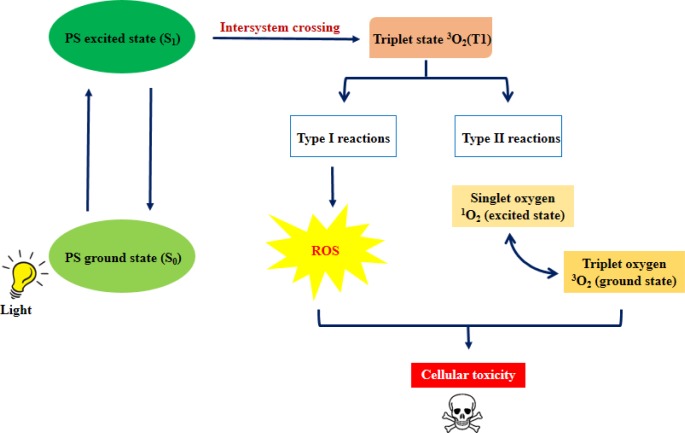
Mechanism of PDT Upon light activation, the photosensitizer is excited (S_0_ to S_1_). S_1_ is converted to a more stable triplet state *via* intersystem crossing. Further, type I reactions involve the formation of ROS, whereas, the loss of energy in type II reactions leads to the formation of highly reactive singlet oxygen species; ultimately leading to cellular toxicity.

Photodynamic therapy (PDT) is a minimally invasive and clinically approved therapy that can be used for early stage disease [7]. PDT involves 3 main components: a photosensitizer, light and tissue oxygen. The basic principle of PDT is that the photosensitizer is activated from its singlet ground state (S_0_) to a short lived excited singlet state (S_1_) upon irradiation with light of appropriate wavelength. The S_1_ can return to S_0_ state by emitting the absorbed energy as fluorescence, or dissipation in the form of heat [8]. Alternatively, S_1_ can convert to a longer lived triplet state (T_1_) *via* intersystem crossing. The energy transfer from T_1_ to biological substrates or molecular oxygen generates reactive oxygen species (ROS) (Figure 1), causing cellular damage leading to death mainly by apoptosis or necrosis [9]. PDT has proven to exert selective cytotoxicity towards malignant cells, leading to cell death [10, 11]. Several photosensitizers that have been studied include hematoporphyrin derivatives (Photofrin), aminolevulinic acid (5-ALA), verteporfin (visudyne), chlorophyll derivatives (pheophorbide a) and more. PDT is frequently used on cutaneous lesions, but has also been tested on cancers of breast [12], lung [13], head and neck [14], esophageal [15, 16], bladder [17] and prostate [18]. Table 1 summarizes the characteristics of different photosensitizers used in PDT, for the treatment of cancer.

**Table 1:** Summary of characteristics of various photosensitizers

Photosensitizer	Mode of action	Activation wavelength	Route of administration	Advantages	Disadvantages
Porfimer sodium (Photofrin)	Tissue based	405 nm	Intravenous	Preparation less heterogenous than HpD derivatives	Prolonged skin photosensitivity; long drug- light intervals; suboptimal tumor selectivity
Aminolevulinic acid (5-ALA)	Tissue based	410 nm	Oral or topical	Selectivity for cancer cells; short drug light interval (up to 4 hours)	Less tissue penetration
Verteporfin	Tissue based	692 nm	Intravenous	Selectivity for cancer cells; short drug light interval (15-30 mins);	Visual disturbances reported
mTHPC (Foscan)	Tissue based	652 nm	Intravenous	Low dose required	Prolonged skin photosensitivity (upto 6 weeks); drug light interval of 3-5 days
Motexafin lutetium (MLu)	Vascular acting	732 nm	Intravenous	Short drug – light interval (3 hours); no reported skin photosensitivity	No disadvantage reported
TOOKAD	Vascular acting	763 nm	Intravenous	Short drug-light interval (mins); no reported skin photosensitivity	No disadvantage reported

## mTHPC

Mesotetra (hydroxyphenyl) chlorin (mTHPC, Foscan) is a single pure chlorine compound, and one of the most potent second generation photosensitizers. Several clinical studies have shown that mTHPC is 100-200 times more potent than Photofrin, which is the most widely used first generation photosensitizer. With an excitation wavelength at 652 nm, it presents a high tumor selectivity. mTHPC is a highly hydrophobic molecule, and this nature ensures its localization in critical intracellular membranous organelles [19].

Several studies have formulated different conjugates of mTHPC to achieve maximal effect. The first commercial liposomal formulation of mTHPC was Foslip. This method involves encapsulating mTHPC in liposomes, which helps decrease the tendency of the photosensitizer to form aggregates thus improving solubility in aqueous media [20]. Petri et al. [21] optimized cellular uptake and photodynamic efficiency of mTHPC using a PEGylated liposomal formulation of mTHPC called Fospeg in PCa cell lines and compared its efficiency to Foscan. The results showed that the intracellular concentration of Fospeg was increased compared to Foscan, with neither demonstrating any dark cytotoxicity. Another group developed ‘Theranosomes’, which are precursor cells (Human macrophages derived from monocytic THP1 cell line), loaded with photosensitizer (mTHPC) and magnetic nanoparticles [22]. They assessed the delivery of mTHPC both *in vitro* (PC3 PCa cells) and *in vivo* (TC-1 murine cervical cancer), followed by PDT treatment. A dose dependent uptake of theranosomes correlated with significant decrease in cell viability post-PDT *in vitro* and tumor size in mice.

The cell death pathways evoked by mTHPC mediated PDT involve oxidative damage which may lead to apoptosis, autophagy or necrosis. Lower doses of photosensitizer have reported apoptosis [23, 24] while higher doses usually lead to autophagy or necrosis [25]. It has been reported that upon PDT treatment there is an increase in ROS which can kill a portion of the cells immediately, while others undergo a death process which takes several hours. mTHPC - PDT was reported to induce immediate DNA damage and reduction of RNA due to ROS production in PC-3 cells. Also, reduced levels of genes involved in cellular defense mechanisms against oxidative and metabolic stress were observed. Furthermore, some HSP70 members were also down regulated [26]. mTHPC - PDT also blocks proliferation and induces cell cycle arrest [27].

As compared to hollow organs, the prostate is a solid organ which makes it challenging for PDT. Several studies have shown necrosis of glandular tissue in hollow organs with little effect on connective tissue. Healing of necrotic tissue in large volumes post PDT is much slower as compared to healing of mucosa in hollow organs [28]. Canine studies of PCa with mTHPC - PDT have shown glandular areas of hemorrhagic necrosis which healed with fibroblast infiltration, but there was still glandular atrophy post 90 days [29]. PDT of prostate can produce areas of rectal mucosal necrosis, however these areas heal with the regeneration of normal mucosa [29].

With its success as a potential therapeutic, mTHPC - PDT has found its way into clinical trials. A study performed at University College London Hospitals assessed mTHPC - PDT in early PCa [28]. The approach did not involve treatment of the whole gland, but only areas of cancer as detected by biopsy. One patient had a period of mild incontinence (4 months) which resolved spontaneously. Another patient experienced reduction in erectile function which was due to right and left neurovascular bundles in the PDT treated areas. A reduction in prostate volume was also observed, whereas PDT studies on normal canine prostate showed necrosis of glandular tissue with preservation of collagen with minimal changes in volume [30]. There was also a significant reduction in PSA levels in the human patients. Another study involved the combination of mTHPC - PDT with fluoropyrinimides, which are used in chemotherapeutic treatment, on PCa cell lines [31]. 5-fluoro-2’-deoxyuridine (5FdUr) was used in this study due to different modes of action of both drugs. mTHPC being lipophilic, accumulates mainly in membranes excluding the nucleus, while 5FdUr acts in the cytosol by inhibiting thymidylate synthase and is incorporated in RNA and DNA [32]. The combination treatment showed higher concentrations of 5FdUr with 0.1μM mTHPC - PDT has an antagonistic effect, while lower concentrations of 5FdUr has an additive effect, with more cell death in comparison to individual treatments.

## TOOKAD

**Figure 2:**
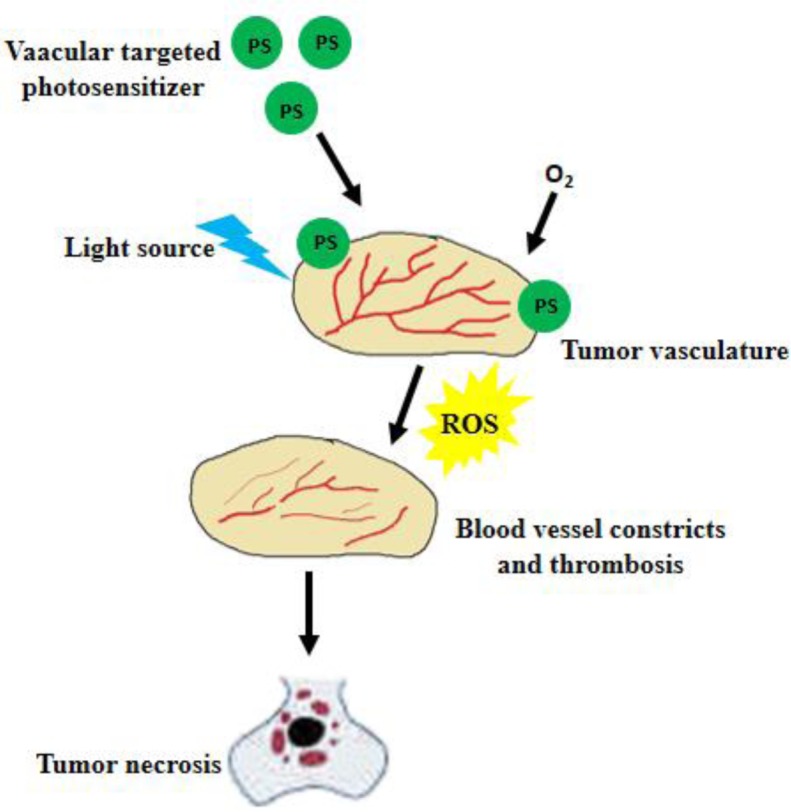
Mechanism of vascular targeted PDT Vascular targeted PS accumulates in the tumor tissue. When light of suitable wavelength activates the PS, ROS is produced leading to vessel constriction, thrombosis and blood stasis; resulting in tumor necrosis.

TOOKAD or WST09 (padoporfin) is a relatively new second generation photosensitizer drug, which is a pure palladium (Pd) - substituted bacteriochlorophyll derivative with a peak absorption wavelength at 763 nm. TOOKAD acts by damaging vasculature and altering blood supply and is generally described as vascular targeted photodynamic therapy (VTP). Damage of vascular endothelium is followed by a series of events, like thrombosis, blood stasis, and vessel occlusion, ultimately leading to tumor necrosis (Figure 2) [33, 34]. It has a relatively fast clearance rate from the body. Several studies have investigated the potential of TOOKAD as a therapeutic to treat PCa, mainly in patients who failed prior radiotherapy.

**Figure 3:**
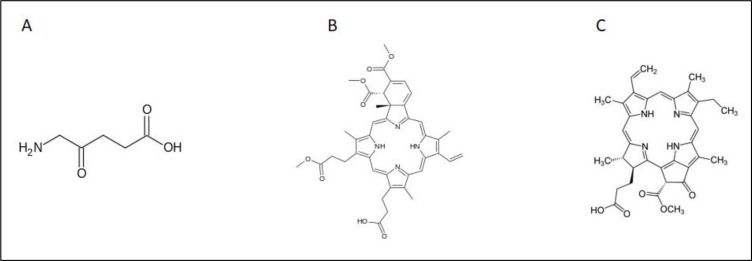
Chemical structures of **A.** 5-ALA, **B.** verteporfin and **C.** pheophorbide a

A phase I study to evaluate the safety of TOOKAD - VTP reported it to be safe with no serious adverse events [35]. A phase II trial was initiated by the same group enrolling 28 patients to study the effects of TOOKAD-VTP with escalating light doses [36]. Patients received a 2 mg/kg dose and light doses were specified by computer-aided treatment planning [37]. The treatment response was assessed by measuring PSA levels, lesion formation (using MRI) and 6-month biopsy. Treatment of the whole prostate was achieved with minimal effects on surrounding organs. They also reported improved treatment efficacy with increased light dose for almost 80% of the prostate in some patients with a decrease in PSA levels. Severe cutaneous photosensitivity is a common problem with PDT drugs, but due to TOOKAD's fast clearance, this is greatly reduced. About 80 % of TOOKAD was cleared from patients within 30 minutes, with negligible concentrations at 150 minutes [38].

The canine prostate has proved to be a good model due to its resemblance both in physical size and in anatomical structure to that of the human prostate. Canine prostate PDT with various photosensitizers has been investigated since the early 1980s [39, 40]. On investigating the effects of TOOKAD PDT on the prostate and surrounding tissues in canine model, hemorrhagic necrosis was observed at one week post TOOKAD PDT in the prostate and prostatic urethra. Mild inflammation was observed on the bladder, colon, abdominal muscle and pelvic plexus [41, 42]. The same group studied effects of TOOKAD-PDT on canine prostate pre-treated with ionizing radiation to produce physiological and anatomical relevance similar to patients for whom radiotherapy has failed. They reported TOOKAD-PDT can safely destroy prostate tissue that has previously received radiation therapy [43]. Another group studied the effects of TOOKAD PDT on peripheral nerve tissue in the saphenous nerve using an *in situ* canine model. It was demonstrated that TOOKAD PDT induced conductivity changes, which depended on both drug and light dose. Treatment with drug alone, light alone, or low dose PDT produced little to no change in nerve conduction properties. Lower light dose (50 J/cm^2^) with a drug dose of 2 mg/kg caused little nerve tissue damage while higher light dose (200 J/cm^2^) and a drug dose of 1 mg/kg caused marked damage to the nerve and surrounding tissue [44].

TOOKAD-PDT effects on human prostate cancer xenograft model in mice with small cell carcinoma of the prostate yielded a cure rate of 69% to 77%. The necrotic process was by vascular damage, leading to complete destruction of the tumor, with consequent tissue remodeling 10 days post PDT. The effects on surrounding tissue were minimal due to the fragile nature of tumor vasculature and the relative resistance of normal vasculature [45]. The same group also reported rapid decline in apparent diffusion coefficient in human prostate adenocarcinoma xenografts which serves as an early response marker for successful TOOKAD-PDT [46].

WST11 (padeliporfin or TOOKAD Soluble) is a second generation photosensitizer of WST09, developed to avoid some encountered subclinical hepatotoxicity in patients with cardiovascular events [47]. WST11 eliminates the Cremophor based formulation to counteract co-solvent effects on blood pressure [48]. Several clinical studies have investigated the effects of WST11 in patients with localized PCa. It proved to be safe and efficient in canines for VTP mediated ablation of large prostatic tissue by vascular occlusion. A study involving 117 men looked at the efficacy of various combinations of WST11 (4 mg/kg) and various light intensities (730 nm and 753 nm). On day 7 post-treatment, the volume of necrosis was reduced to 76.5% with 68.4% of patients demonstrating negative biopsies by 6 months [49]. Even though focal therapies with TOOKAD-VTP are promising, they are not the standard for men with organ confined PCa [50]. A study reported the oncologic and functional outcomes to assess the feasibility, safety and efficacy of salvage radical prostatectomy post TOOKAD-VTP [51]. It was deemed easy for patients who received a unilateral treatment, whereas bilateral treatment induced peri - prostatic fibrosis leading to difficulties in the dissection of posterior and lateral sides of the prostate. Many groups have applied techniques to optimize and analyze VTP. The dose of light is more important for successful PDT treatment than the amount of energy delivered [52].

A phase II study with 42 men by Moore et al. [53] determined optimal drug and light dose to achieve focal ablation using WST11. Biopsy data and post treatment MRI data indicated 4 mg/kg WST11 with 200 J/cm energy were optimal treatment conditions. Day 7 MRI results showed a negative biopsy rate of 31% in the 16 men who received 4 mg/kg WST11. In another study involving 56 patients, histopathology of prostate before and 6 months post WST11-VTP was studied [34]. Areas subjected to VTP were recognized as well demarcated hyaline fibrotic scars, with or without coagulative necrosis. Mild to moderate chronic inflammation was also observed with few atrophic benign glands, or corpora amylacea surrounded by multinuclear macrophages.

## MOTEXAFIN LUTETIUM

Motexafin Lutetium (MLu) is a second generation photosensitizer with a tripyrrolic pentaaza-expanded porphyrin and an absorption wavelength of 732 nm. MLu has reported efficacy in several murine tumor models, canine models and human clinical trials. In a study on effects of MLu and PDT delivery methods in normal canine prostate model, it was found that MLu - PDT initially caused inflammation and necrosis, followed by glandular atrophy and fibrosis [54].

A Phase I clinical trial was initiated at the University of Pennsylvania in patients with recurrent prostate carcinoma [55]. MLu was administered intravenously (0.5 - 2 mg/kg) at various times prior to light delivery. On measuring MLu concentrations and optical properties of human prostates, they found substantial inter and intra patient heterogeneity. Also, the mean light penetration (732 nm) of the human prostate was found to be 0.4 cm, which is two times smaller than the canine prostate [56]. Another study by Du et al. [57] demonstrated safe and comprehensive treatment of PCa using PDT in canines and humans alike. However, a significant dose distribution variability was observed along with a rise in PSA levels post treatment. Other studies also show intra- and inter- patient heterogeneity in optical properties of MLu in the prostate pre- and post- PDT [58–60].

Since PSA levels correlate to diagnosis of PCa, PSA levels pre- and post- PDT can help measure the effectiveness of treatment. For instance, rise in PSA correlates to the invasiveness of the procedure [61]. Also, PSA levels are detected over a period ranging from few hours to several weeks after treatment [62–64]. MLu - PDT causes a short term increase in serum PSA levels, which may be due to cellular damage, leading to release of PSA in circulation [64]. The extent of PSA reduction subsequent to its initial therapy induced spike elicits the biochemical success of PDT on prostate and may be a useful indicator of treatment efficacy [65]. Hence, the change in PSA is related to PDT dose, as well as PS concentration, in tissue [66].

As PDT requires oxygen for tumor damage, it is essential to study the hemodynamic responses, which mainly are tumor blood flow and tumor oxygen saturation. Pogue et al. hypothesized that treatment efficiency depends on tumor oxygenation during PDT, and under oxygen limiting conditions, treatment efficiency can be repealed [67]. A preliminary study on three patients found that total hemoglobin concentration (THC) and blood flow decreased during MLu - PDT along with a slight decrease tumor blood oxygen saturation [68].

## AMINOLEVULANIC ACID

**Figure 4:**
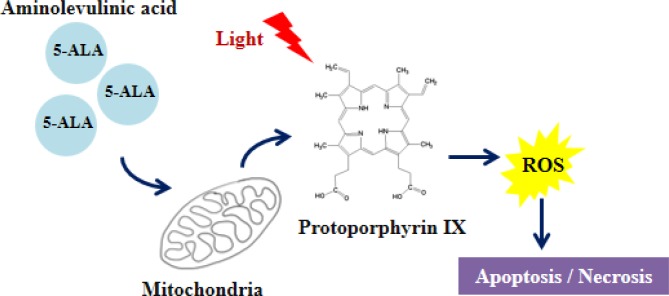
5-ALA-PDT induces cell death *via* apoptosis/necrosis 5-ALA accumulates in the mitochondria, and forms protoporphyrin IX using the heme synthesis pathway. Activation with light of specific wavelength causes a photodynamic reaction, producing ROS, which in turn leads to cell death.

Aminolevulanic acid (5-ALA), a pro-drug, which is a biosynthetic precursor of the photosensitizer protoporphyrin IX [69], has led to many applications in which 5-ALA or ALA esters can be administered topically or orally (Figure 3A). PDT using 5-ALA (Figure 4) has been extensively studied in the treatment of premalignant and malignant skin tumors [70, 71]. The introduction of topically applied 5-ALA [72] demonstrated complete response rates for non-hyperkeratotic actinic keratosis lesions exceeding 75%. 5-ALA-PDT has also shown promising results for the treatment of superficial and nodal basal cell carcinoma [73–75] (Table 2). Unfortuately, the use of 5-ALA-PDT to treat prostate cancer is a little more mixed. Using the Dunning Rat R3327 tumor model, the intravenous application of 5-ALA with subsequent irradiation using 633nm laser resulted in 97% necrosis in one study [76] and highly variable results in another due to hypoxic conditions and poor 5-ALA distribution in tumor [77]. PDT application to normal prostate tissue in dogs resulted in small lesions suggesting weak distribution in tissue as well [78]. More *in vivo* studies are needed to assess 5-ALA-PDT as a clinical treatment for prostate cancer. However, 5-ALA has gained most of its recognition in the prostate field for its use in photodynamic diagnosis (PDD). Nakai et al. incubated 5-ALA with voided urine samples subsequent to prostate massage, which produced protoporphyrin IX in shed prostate cells, resulting in a 74.1% sensitivity and 70.2% specificity levels. This demonstrated higher sensitivity than both abnormal digital rectal exam and transrectal ultrasound, and more specific than PSA levels [79]. A stomach tube delivered 5-ALA orally in another study and PPD labelled cells during a radical prostatectomy resulted in 75% sensitivity and 87% specificity [80]. Early studies support potential for clinical use in diagnostics but more randomized clinical trials are needed to confirm its use.

**Figure 5:**
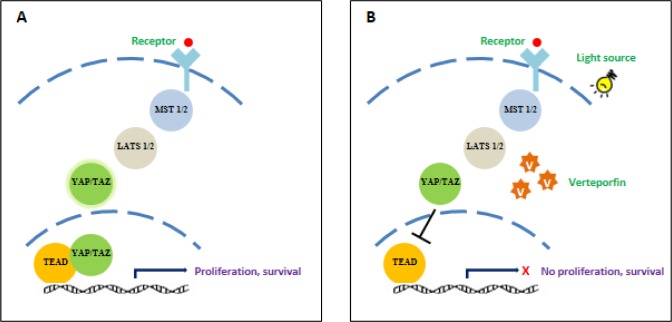
Verteporfin inhibits the Hippo signaling pathway, blocking cell proliferation and survival **A**. Under normal conditions, growth signals activate the Hippo signaling pathway, causing the activation of YAP/TAZ complex. This complex translocates into the nucleus to form YAP/TEAD which activates several growth factors, leading to cell growth and proliferation. **B**. Verteporfin blocks the translocation of YAP/TAZ into the nucleus, thus inhibiting cell growth and survival.

**Table 2:** PDT in clinical trials

Photosensitizer	Clinical trial phase (ClinicalTrials.gov)		
	Phase I	Phase II	Phase III
Porfimer sodium (Photofrin)	Pancreatic cancer	Human head & neck cancer Cholangiocarcinoma	Esophageal and/or gastric cardiac cancer
Aminolevulinic acid (5-ALA)	Early stage head & neck tumors Multple basal cell carcinomas Colon cancer	Malignant gliomas Basal cell carcinoma	
Verteporfin	Brain tumors	Age – related macular degeneration	
mTHPC (Foscan)	Non-small cell lung cancer	Nasopharyngeal carcinoma Cholangiocarcinoma	
TOOKAD	Renal tumors		

## VERTEPORFIN

Verteporfin, a benzoporphyrin derivative (Figure 3B) is commonly used as a PS for PDT to eliminate abnormal blood vessels in the eye, such as the wet form of age related macular degeneration [81]. Recently, verteporfin has gained recognition in VTP and its action is known to be mediated by the Hippo signaling pathway, which controls organ size by the regulation of cell cycle, proliferation and apoptosis [82]. The activity of Yes-associated protein (YAP) is essential in Hippo signaling, and high levels of YAP have been observed in hepatocellular carcinoma [83]. The interaction of YAP with transcription enhancers activation domain (TEAD) family, upregulates the expression of various growth factors including connective tissue growth factor (CTGF) and Cyr61 [84], AXL receptor tyrosine kinase [85], survivin and c-myc [86]. A study on the effects of verteporfin on retinoblastoma reported that verteporfin induces growth inhibition, apoptosis and cell cycle arrest by interfering with the YAP-TEAD growth pathway (Figure 5) [87].

Nguyen et al. [88] studied the significance of ERG in human prostate cancer and determined that ERG binds to the YAP1/TEAD region, leading to the activation of Hippo target genes. As mentioned above, verteporfin is an inhibitor of YAP-TEAD growth pathway, it has a therapeutic potential for prostate cancer. MatLyLu tumors in rat models were sensitive to verteporfin-PDT regimen inducing tumor necrosis [89]. Furthermore, VTP with verteporfin led to a dose and time-dependent increase in vascular permeability and decrease in blood perfusion in EGFP-MatLyLu prostate tumor cells [90].

## PHEOPHORBIDE

Pheophorbide a (Pba), a breakdown product of chlorophyll *a* (Figure 3C), can be derived from algae and higher plants [91]. Early studies comparing the photodynamic efficacy of Pba with Hematoporphyrin derivative (HpD) in Lewis lung carcinoma in mice [92] found that Pba is a stronger PS as compared to HpD due to its longer wavelength of absorbance in the red region of the spectrum. Another study suggesting the therapeutic potential of Pba-PDT studied rat pituitary tumor implanted in nude mice [93]. Pba administered intravenously accumulated in the tumor, and significant reduction in tumor mass was observed upon irradiation. Pba-PDT has also been tested *in vivo* and *in vitro* in human pancreatic cancer [94, 95]. Inhibition in cell growth was observed (0.5 μM and 2 μM concentrations of Pba), along with establishing apoptosis due to DNA fragmentation as the mode of cell death. Another group studying the effects of Pba-PDT on hepatocellular carcinoma found that Pba accumulates in the mitochondria. Upon photoactivation, Pba induced membrane deterioration, causing release of cytochrome c into the cytoplasm, thus activating the intrinsic apoptotic pathway (Figure 7) [96, 97]. Pba-PDT has also shown promising therapeutic potential in breast cancer [98], bladder cancer [17], prostate cancer [18, 99] and esophageal cancer [100].

## HEMATOPORPHYRIN DERIVATIVE

**Figure 6:**
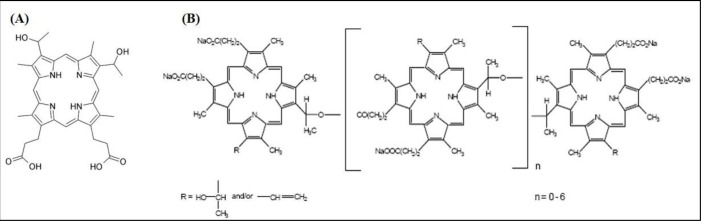
Structural difference between A. hematoporphyrin and B. Photofrin

Hematoporphyrin derivative (HpD) is a complex mixture of monomeric and aggregated porphyrins derived from hematoporphyrin. HpD has been used successfully for localization and photoradiation therapy of tumors [65]. HpD was first used in 1960 by Lipson and Baldes [101] in mice. The first use of HpD in humans to treat a patient with breast cancer occurred in 1966 [102] and since then, several studies on different cancers have been reported. Several groups reported the use of HpD in the treatment of tumors in animals [103], and in patients with bladder cancer [104], head and neck cancer [105, 106]. In 1984, Dougherty et al. [107] fractionated HpD to examine and isolate the active compounds, which were found to be multiple porphyrin ethers and esters (Figure 6). This mixture was marketed in 1993 as Photofrin (sodium porfimer) and was approved in Canada for the treatment of early bladder cancer. The United States Food and Drug Administration approved sodium porfimer for the treatment of esophageal cancer in 1996 [108], following approval in France and The Netherlands for the treatment of advanced lung and esophageal cancer; in Germany for the treatment of early lung cancer; and Japan for the treatment of early esophageal, lung, gastric and cervical cancer [109].

**Figure 7:**
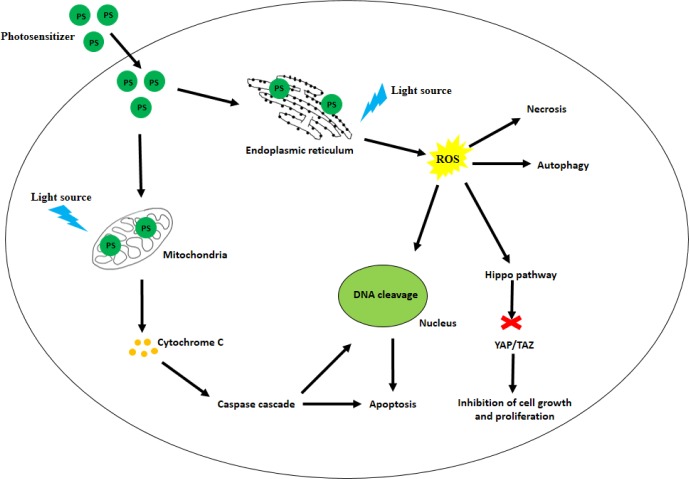
Various cell death pathways modulated by PDT The PS are taken up by the malignant cells and populate in various organelles like mitochondria and the endoplasmic reticulum. Light of suitable wavelength activates the PS which in turn causes release of cytochrome C from the mitochondria; leading to the activation of the caspase cascade. Pro-apoptotic factors like p53 are activated, thus inducing apoptosis. Formation of ROS may also lead to necrosis or autophagy. As shown earlier, the Hippo signaling pathway can be inhibited leading to inhibition in cell growth.

## OTHER PHOTOSENSITIZERS

One of the limitations of conventional PDT is poor tissue penetration of the PS due to self-aggregation rendering it unable to permeate the lipid bilayer, leading to reduced efficacy [110, 111]. There has been a constant search for improved formulations so as to make the PS easily deliverable and highly efficacious. One such approach applies the use of nanoparticles, which can protect the PS from recognition and clearance from the biological system prior to reaching the target [112]. Upon testing polymethylmethacrylate core-shell fluorescent nanoparticles loaded with aluminium pthalocyanine (Ptl) in human prostate tumor model, Duchi et al. demonstrated that nanoparticles with Ptl significantly reduced tumor growth by 75%, whereas Ptl alone could reduce tumor growth by 50% [113]. Similarly, uptake of gold nanoparticles loaded with verteporfin in HeLa was increased (98.6%) as compared to free verteporfin (18.86%) [114]. The use of different sizes of submicron magnetic particles in PC-3 prostate cancer cells to assess photodynamic anti-cancer activity showed successful reduction in cell viability in a size dependent manner, and the anti-cancer activity depends on the concentration inversely proportionate to particle size [115].

Along with therapeutic efficiency, PS can also be used for imaging and diagnosis of cancerous tumors. PS-based fluorescence imaging has shown promising results in the detection of ovarian cancer [116, 117], pancreatic cancer [118, 119] and lung cancer [120, 121].

## CONCLUSIONS

PDT has potential as a focal treatment for PCa. Several clinical trials using vascular targeted photosensitizers have established it as safe, effective, feasible and repeatable. Since the first use of PDT for PCa in a clinical setting in 1990, several advances in photosensitizer design and light delivery have been achieved. Heterogeneity of response, tissue light penetration and tissue oxygenation are current limitations, which can be overcome with further studies. Potential advances in photosensitizer and light delivery, along with treatment monitoring systems, will make PDT an exciting addition to the array of treatments available for PCa in primary and post radiotherapy setting.

## FUTURE OF PDT IN PCa TREATMENT

Photodynamic therapy for PCa can potentially be very selective and a single session treatment, as well as it can be used in primary or salvage settings. Even though photosensitivity and phototoxicity are important factors associated with PDT, researchers are attempting to establish optimum treatment parameters involving intraprostatic drug levels, light source and tissue oxygen. With the advent of new PS that specifically target cancerous tissue with minimal phototoxicity in normal tissue, the future of PDT has demonstrated promise. For PCa therapy, the light should be able to penetrate deeper under the skin through the body tissue. Hence, improvement in light delivery equipment is also being constantly improved. So far, the clinical trial data for PCa looks promising, and this therapy will greatly benefit men with early and advanced PCa.
